# Estimating Mortality Derived from Indoor Exposure to Particles of Outdoor Origin

**DOI:** 10.1371/journal.pone.0124238

**Published:** 2015-04-10

**Authors:** Wenjing Ji, Bin Zhao

**Affiliations:** Department of Building Science, School of Architecture, Tsinghua University, Beijing, 100084, China; The Ohio State University, UNITED STATES

## Abstract

Following an extensive review of the literature, we further analyze the published data to examine the health effects of indoor exposure to particulate matter (PM) of outdoor origin. We obtained data on all-cause, cardiovascular, and respiratory mortality per 10 μg/m^3^ increase in outdoor PM_10_ or PM_2.5_; the infiltration factors for buildings; and estimated time spent outdoors by individuals in the United States, Europe, China, and globally. These data were combined log-linear exposure–response model to estimate the all-cause, cardiovascular, and respiratory mortality of exposure to indoor PM pollution of outdoor origin. Indoor PM pollution of outdoor origin is a cause of considerable mortality, accounting for 81% to 89% of the total increase in mortality associated with exposure to outdoor PM pollution for the studied regions. The findings suggest that enhancing the capacity of buildings to protect occupants against exposure to outdoor PM pollution has significant potential to improve public health outcomes.

## Introduction

Associations between exposure to particulate matter (PM) pollution and increased morbidity and mortality have been observed in both population-based [1–3] and cohort-based [4–9] research. PM is associated with negative health impacts, including cerebrovascular disease (stroke), respiratory infections, cardiopulmonary disorders, ischemic heart disease, and lung cancer [10–16]. Weichenthal et al. [17] examined the relationship between PM_2.5_ and non-accidental and cardiovascular mortality in the U.S. Agricultural Health Study cohort. Rural PM_2.5_ exposure may be associated with cardiovascular mortality in men; however, similar associations were not observed among women. Chen et al. [18] and Brook et al. [19] conducted population-based cohort studies, which indicated that long-term exposure to PM_2.5_ is associated with increased risk of mortality attributable to diabetes. Burnett et al. [13] developed a fine particulate mass-based relative risk model for estimating the global burden of disease attributable to ambient exposure to fine particulate matter, which was found to be a superior predictor of relative risk (RR) compared with seven other forms previously used in burden assessments. Wu et al. [14] examined the cardiopulmonary health effects of PM_2.5_ from different pollution sources in China. They repeatedly examined for a series of cardiopulmonary health indicators among a panel of 40 healthy university students, while simultaneously collecting daily ambient PM_2.5_ mass samples and measuring for 29 chemical constituents in the laboratory throughout the study. Their results indicated that different sources of PM_2.5_ may play important roles in different aspects of PM_2.5_ related cardiopulmonary health effects.

However, a limitation of most of these studies is that only outdoor PM pollution exposure was measured. In many buildings, high concentrations of ambient PM pollution enter the indoor environment [20–23], where people spend approximately 90% of their time [24]. There are two scenarios for personal exposure to outdoor PM pollution. The first is that individuals are directly exposed while outdoors. The second is that people remain indoors, but are exposed to particles that enter the building by means of infiltration or ventilation. The infiltration factor, defined as the fraction of the outdoor concentration that penetrates indoors and remains suspended, is a potential source of exposure variation but is often overlooked in epidemiological studies [25]. Infiltration depends on the air exchange rate, PM loss rate (the rate at which PM is removed from the air by deposition, filtration, and so forth), and penetration efficiency (the fraction of PM that penetrates the building envelope as outdoor air moves indoors) [26–27].

Many studies have examined the association between outdoor particles and mortality, and these existing epidemiological data are implicitly influenced by the fact that people spend approximately 90% of their time [24] indoors. Moreover, previous studies did not differentiate outdoor exposure from indoor exposure to particles of outdoor origin. Wilson et al. [28] discussed the legal and scientific importance of assessing personal exposure in terms of ambient particles outdoors and the fraction that infiltrates indoors. This discrete assessment of indoor exposure to indoor particles of outdoor origin is especially important for developing appropriate strategies for controlling indoor air quality. Therefore, the present study focuses on indoor exposure to particles of outdoor origin.

Although Wilson et al. [28] pointed out that it is important to estimate mortality associated with indoor exposure to particles of outdoor origin, to date, no such studies have been presented. For the purpose of controlling aerosol pollution, we are eager to learn from the epidemiological data, e.g. mortality derived from indoor exposure to particles of outdoor origin, in order to support strategies for managing indoor air quality (IAQ).

The study utilizes existing epidemiological data on the mortality of outdoor PM, combined with a physical model of aerosol mechanisms, to estimate mortality associated with indoor exposure to PM of outdoor origin. To the best of our knowledge, this is the first attempt to quantify this relationship for PM exposure.

## Methods

### Analytical model

Most epidemiological studies on the health effects of PM pollution use increased mortality or hospital admissions per 10 μg/m^3^ increase in PM exposure as the health endpoint [6–7, 29–31]. Daniels et al. [29] indicated that log-linear models are appropriate for assessing the effect of PM pollution on daily mortality. They examined the hypothesis of linearity in relation to PM-mortality by comparing the Akaike information criterion (AIC) values obtained under the linear-, threshold-, and spline dose-response models. Their results indicated that a log-linear model is preferable to the threshold and spline models, and so a log-linear analytical model is used here. This approach involves several assumptions: a) Outdoor air pollution is not affected by indoor sources; b) There are no interactions with indoor sources, including allergens and various chemicals; c) The health effects of particle exposure are a function of PM_2.5_ mass concentration. The toxicity of PM_2.5_ is assumed to differ only with mass exposure and not with PM_2.5_ composition [13, 32–33]. Based on these assumptions, the log-linear analytical model used for estimating the health effects of indoor exposure to outdoor-originated PM can be represented as:

$$\Delta \log M_{in,j}=\frac{\Delta C_{out-in}t_{in}}{\Delta C_{out}t_{out}+\Delta C_{out-in}t_{in}}\Delta \log M_{all,j}$$ (1)

Where Δlog *M*_*all*, *j*_ is the increase in mortality due to the j^th^ outcome associated with total PM exposure for each 10 μg/m^3^ increase in PM_10_ or PM_2.5_ outdoors. j represents three major health outcomes: all-cause, cardiovascular, and respiratory mortality.

Δ*C*_*out*_ is the increase in outdoor PM_10_ or PM_2.5_ concentrations, which is set as 10 μg/m^3^.

Δ*C*_*out-in*_ is the increase in outdoor-originated PM_10_ or PM_2.5_ concentrations found in the indoor environment.

*t*_*out*_ is the duration of direct exposure to outdoor PM pollution.

*t*_*in*_ is the duration of indoor exposure to PM of outdoor origin.

Δlog *M*_*in*, *j*_ estimates the increase in mortality due to the j^th^ outcome associated with indoor exposure to outdoor-origin PM for each 10 μg/m^3^ increase in PM_10_ or PM_2.5_. A relatively low value of Δlog *M*_*in*, *j*_ suggests low probability of morbidity or mortality. This implies that buildings adequately shield occupants against outdoor-origin PM pollution and, thus, investment in further reducing the indoor concentrations of such PM would have minimal benefits for public health outcomes. However, a high value of Δlog *M*_*in*, *j*_ suggests high possibility of morbidity or mortality with increased exposure to outdoor-origin PM, emphasizing the importance of preventing ambient PM from entering the indoor environment.

When an epidemiologic study is performed, the observed mortality rate is lower than that in other places with higher infiltration factors, and this can be falsely interpreted that the dose-response slope (Δlog *M*_*in*, *j*_) is less steep. When this mortality estimate was combined with the lower exposure estimate (due to low infiltration) in Eq (1), we would get too low estimation for the mortality due to indoor exposure to outdoor PM. Therefore, the local infiltration factor should be used to adjust the observed mortality rate. We assume that the logarithms of the observed probabilities (or rates) of disease have the probability distributions as:

log(M)=a+bC (2)

Where *M* is the probability of disease, *a* is a constant describing the background probability, *b* is a risk coefficient for the exposure, and *C* is the exposure concentration in the population.

The differences in the probability of disease are caused by the differences in exposure *C*. The observed *C* itself (the actual exposure concentration) is only the surrogate *C*_*obs*_, which in this case is the outdoor concentration of PM *C*_*out*_. With a given difference in the probability of disease between the exposed and non-exposed groups, a biased probability of disease could be described as:

$$\log(M_{E})-\log(M_{0})=(a+bC_{E})-(a+bC_{0})=b(C_{E}-C_{0})=b_{\text{obs}}(C_{E,obs}-C_{0,obs})$$ (3)

where *E* is the exposed group, *0* is the non-exposed group, and *obs* is the biased observed variable (in contrast to the actual variable we would observe if all measurements were correct). Then the ratio of the biased and correct risk estimates is:

$$\frac{b_{obs}}{b}=\frac{C_{E}-C_{0}}{C_{E,obs}-C_{0,obs}}=\frac{\sum_{i}C_{E,i}t_{i}-\sum_{i}C_{0,i}t_{i}}{C_{E,out}\cdot 24-C_{0,out}\cdot 24}$$ (4)

where *i* means different microenvironments and *t*_*i*_ is the time spent in each microenvironment. Infiltration (*F*_*i*_) was used to denote the relative exposure concentrations in different microenvironments *I* (in this case of only indoor and outdoor microenvironments, *F*_*i*_ is 1 for outdoor and equal to infiltration factor (*F*_inf_) for indoor), then

$$\frac{b_{obs}}{b}=\frac{\underset{i}{\sum }F_{i}C_{E,out}t_{i}-\underset{i}{\sum }F_{i}C_{0,out}t_{i}}{C_{E,out}\cdot 24-C_{0,out}\cdot 24}=\frac{(C_{E,out}-C_{0,out})\underset{i}{\sum }F_{i}t_{i}}{(C_{E,out}-C_{0,out})\cdot 24}=\frac{\underset{i}{\sum }F_{i}t_{i}}{24}$$ (5)

The observed *b* is biased downward if the population spends a lot of time in microenvironments with low infiltration factor. The Δlog *M*_*all*, *j*_ can be calculated as follows:

$$\Delta \log M_{all,j}=\frac{24\cdot \Delta \log M_{obs,j}}{\underset{i}{\sum }F_{i}t_{i}}$$ (6)

Combine the Eq (1) and Eq (6), we can get:

$$\Delta \log M_{in,j}=\frac{\Delta C_{out-in}t_{in}}{\Delta C_{out}t_{out}+\Delta C_{out-in}t_{in}}\cdot \frac{24\cdot \Delta \log M_{obs,j}}{\underset{i}{\sum }F_{i}t_{i}}$$ (7)

### Epidemiological data

The epidemiological data for all-cause, cardiovascular, and respiratory mortality attributable to outdoor PM exposure (PM_10_ or PM_2.5_) are based on meta-analyses published in the U.S., Europe, China, and globally between 2000 and 2012. All of the parameters used in the model are summarized in Table 1.

**Table 1:** Parameters used to evaluate of the effects on mortality of indoor exposure to particulates of outdoor origin.

	Location	Reference	Values	Remarks
$\Delta \log M_{obs,j}$	Overall world	Anderson et al. [31]	0.9% (0.6%, 1.3%)	Mean (95%CI) all-cause mortality; PM_2.5_.
			1.3% (0.5%, 2.2%)	Mean (95%CI) cardiovascular mortality; PM_2.5_.
			1.1% (0.2%, 2.0%)	Mean (95%CI) respiratory mortality; PM_2.5_.
	United States	Daniels [29]	0.54% (0.33%, 0.76%)	Mean (95%CI) all-cause mortality; PM_10_.
		Zanobetti and Schwartz [34]	0.98% (0.75%, 1.22%)	Mean (95%CI) all-cause mortality; PM_2.5_.
			0.85% (0.46%, 1.24%)	Mean (95%CI) cardiovascular mortality; PM_2.5_.
			1.68% (1.04%, 2.33%)	Mean (95%CI) respiratory mortality; PM_2.5_.
	Europe	Anderson et al. [31]	0.6% (0.4%, 0.8%)	Mean (95%CI) all-cause mortality; PM_10_.
			0.9% (0.5%, 1.3%)	Mean (95%CI) cardiovascular mortality; PM_10_.
			1.3% (0.5%, 2.0%)	Mean (95%CI) respiratory mortality; PM_10_.
	China	Chen et al. [35]	0.35% (0.18%, 0.52%)	Mean (95%CI) all-cause mortality; PM_10_.
			0.44% (0.23%, 0.64%)	Mean (95%CI) cardiovascular mortality; PM_10_.
			0.56% (0.31%, 0.81%)	Mean (95%CI) respiratory mortality; PM_10_.
		Cao et al. [36]	0.20% (0.1%, 0.3%)	Mean (95%CI) all-cause mortality; PM_2.5_.
			0.3% (0.1%, 0.40%)	Mean (95%CI) cardiovascular mortality; PM_2.5_.
			0.4% (0.2%, 0.6%)	Mean (95%CI) respiratory mortality; PM_2.5_.
$\Delta C_{out}$			10 μg/m^3^	Each 10 μg/m3 increased in outdoor PM_10_ or PM_2.5_.

We selected meta-analyses by Anderson et al. [31] that formed part of the World Health Organization’s “Systematic Review of Health Aspects of Air Pollution in Europe” project. The data were drawn from time-series (ecological and individual) estimates of the effects of PM_10_ on all-cause mortality in 33 European cities or regions. The majority of these estimates originated from multi-city studies conducted in France, Italy, and Spain in 2003–2004. The European exposure–response coefficient for all-cause mortality was 0.6% (95% CI: 0.4%, 0.8%), referring to the percentage change in the number of deaths with each 10 μg/m^3^ increase in outdoor PM_10_. The corresponding summary estimates for cardiovascular and respiratory mortality were 0.9% (95% CI: 0.5%, 1.3%) and 1.3% (95% CI: 0.5%, 2.0%) respectively.

The meta-analysis of Anderson et al. [31] was used to estimate all-cause, cardiovascular, and respiratory mortality attributable to outdoor PM_2.5_ exposure for the U.S., Canada, and globally in 2003–2004. Studies from North and South America as well as other areas of the world were identified in the database and used to conduct meta-analyses for each mortality group. The global exposure–response coefficients for all-cause, cardiovascular and respiratory mortality for a 10 μg/m^3^ increase in outdoor PM_2.5_ were 0.9% (95% CI: 0.6%, 1.3%), 1.3% (95% CI: 0.5%, 2.2%), and 1.1% (95% CI: 0.2%, 2.0%), respectively.

We used results from Daniels et al. [29] to estimate all-cause mortality due to outdoor PM_10_ exposure in the U.S. Their analysis used a database developed for the “National Morbidity, Mortality, and Air Pollution Study” for the 20 largest metropolitan areas in the U.S. over a 7-year period (1987–1994). These data were obtained from the Aerometric Information Retrieval System database maintained by the U.S. Environmental Protection Agency (EPA). Their estimates for all-cause, mortality with a 10 μg/m^3^ increase in outdoor PM_10_ in the U.S. were 0.54% (95% CI: 0.33%, 0.76%).

In the U.S., Zanobetti and Schwartz [34] conducted a national, multi-city time-series study of the acute effects of PM_2.5_ on risk of death from all causes, cardiovascular disease, myocardial infarction, stroke, and respiratory mortality for the years 1999–2005. They found a 0.98% increase (95% CI, 0.75%, 1.22%) in total mortality, a 0.85% increase (95% CI, 0.46%, 1.24%) in cardiovascular deaths, and a 1.68% increase (95% CI, 1.04%, 2.33%) in respiratory deaths for a 10 μg/m^3^ increase in two-day averaged PM_2.5_.

After reviewing studies published in both English and Chinese, on the health effects of PM_10_ in China, we chose the meta-analysis conducted by Chen et al. [35], on the association between PM_10_ and daily mortality in 16 Chinese cities between 1996 and 2008. Their results showed exposure–response coefficients of 0.35% (95% CI: 0.18%, 0.52%), 0.44% (95% CI: 0.23%, 0.64%), and 0.56% (95% CI: 0.31%, 0.81%) for all-cause, cardiovascular, and, respiratory mortality, respectively, resulting from a 10 μg/m^3^ increase in outdoor PM_10_ in China.

For China, we used results from Cao et al. [36] on the short-term association between PM_2.5_ constituents and daily mortality in Xi’an, a heavily polluted Chinese city. Those authors obtained daily mortality data and daily concentrations of PM_2.5_ for 1 January 2004 through 31 December 2008. Their results show that the exposure–response coefficients for all-cause, cardiovascular, and respiratory mortality for a 10 μg/m^3^ increase in outdoor PM_2.5_ in China were 0.20% (95% CI: 0.1%, 0.3%), 0.3% (95% CI: 0.1%, 0.4%), and 0.4% (95% CI: 0.2%, 0.6%), respectively.

### Outdoor-originated particles in the indoor environment

The infiltration factor, *F*_inf_, is used to determine the relationship between outdoor PM concentration and indoor PM derived from outdoor sources.

The indoor PM_2.5_ concentration can be calculated as:

$$\frac{dC_{in}}{dt}=\lambda pC_{out}-(\lambda +\beta_{PM})C_{in}+\dot{S}$$ (8)

where *C*_*in*_ is indoor PM mass concentration (μg/m^3^), *C*_*out*_ is outdoor PM mass concentration (μg/m^3^), *p* is PM penetration rate, *λ* is hourly air exchange rate (h^-1^), *β*_*PM*_ is particle deposition rate (h^-1^), and $\dot{S}$ is the volume-averaged indoor PM_2.5_ source strength (μg/(h•m^3^)).

According to the steady-state assumption [37], the rates of penetration (*P*), deposition (*β*_*PM*_), and air exchange (*λ*) remain constant over a given time. Eq (8) can then be solved as:

$$C_{in}=\frac{\lambda \cdot p}{\left(\lambda +\beta_{PM}\right)}C_{out}+\frac{\dot{S}}{\left(\lambda +\beta_{PM}\right)}$$ (9)

The first item on the right side of Eq (9) represents the contribution of outdoor-originating particles, which is the infiltration definition, and the second item represents the contribution of indoor-emitted particles.

Therefore, infiltration factor is defined as:

$$F_{\inf}=\frac{\lambda \cdot p}{\text{(λ+}\beta_{PM})}$$ (10)

The infiltration factor for PM_2.5_ is typically higher than that of PM_10_, due to the stronger effect of the deposition mechanism on gravity setting for coarse particles, which ultimately results in greater particle loss in the cracks of building envelopes and on indoor surfaces for PM_10_. Furthermore, since the infiltration factor is a function of a building’s crack geometry as well as its air exchange rate, particle deposition rate and penetration factor (the fractional penetration of particles from outdoors), the mean infiltration factors measured in these studies show considerable variation.

The literature was reviewed to determine the PM_10_ and PM_2.5_ infiltration factors reported for European, North American, Chinese, and global residences (see Table 2). We calculate mean infiltration factors of PM_10_ and PM_2.5_ in each region, and the maximum and minimum values for each region were also used in sensitivity analysis to study the effect of infiltration factor on mortality and particle infiltration (see Table 3). For the PM_2.5_ infiltration in China, there is no available data in literature, therefore, we calculated it according to the Eq (4). The maximum infiltration air exchange rate was 0.55 h^-1^ and the minimum infiltration air exchange rate was 0.06 h^-1^ according to Zhou and Zhao [44] study of Beijing region. The PM_2.5_ penetration rate was 0.8 and the deposition rate of PM_2.5_ was 0.09 h^-1^according to the systematic review by Chen and Zhao [26]. Therefore, the PM_2.5_ infiltration in China was set 0.69 (maximum) and 0.32 (minimum).

**Table 2:** Review of infiltration factors for PM_10_ and PM_2.5_ in the United States, Europe, and China.

	Reference	Values	Location
PM_10_ infiltration	Ozkaynak et al. [38]	0.51	Riverside. USA
		0.52	Riverside. USA
	Ozkaynak et al. [39]	0.60	Riverside. USA
	Ott et al. [40]	0.55	Riverside. USA
	Lazaridis et al. [41]	0.45	Oslo. Norway
	Hoek et al. [42]	0.17	Helsinki, Finland
		0.28	Athens, Greece
		0.41	Amsterdam, Netherlands
		0.27	Birmingham, UK
	Diapouli et al. [43]	0.56	Athens, Greece
	Zhou and Zhao [44]	0.33	Anshan
		0.34	Beijing
		0.42	Fuzhou
		0.42	Guangzhou
		0.38	Hangzhou
		0.45	Hong Kong
		0.29	Lanzhou
		0.38	Shanghai
		0.30	Shenyang
		0.37	Suzhou
		0.31	Taiyuan
		0.33	Tangshan
		0.34	Tianjin
		0.30	Urumqi
		0.37	Wuhan
		0.35	Xi'an
PM_2.5_ infiltration	Ozkaynak et al. [38]	0.70	Riverside. USA
		0.56	Riverside. USA
	Lee et al. [45]	0.62	Chongju. Korea
	Lachenmyer and Hidy [46]	0.66	Birmingham. USA
	Wallace et al. [47]	0.48	Seven cities. USA
	Williams et al. [47]	0.45	North Carolina. USA
	Reff et al. [48]	0.51	Three cities. USA
	Wallace and Williams [49]	0.55	North Carolina. USA
	Sarnat et al. [23]	0.48	L.A. USA
	Hoek et al. [42]	0.63	Three Cities, USA
	Ozkaynak et al. [39]	0.71	Riverside. USA
	Polidori et al. [50]	0.47	Los Angeles
	Allen et al. [27]	0.62	USA
		0.47	USA
		0.82	USA
	Meng et al. [51]	0.56	USA
	Haonninen et al. [52]	0.70	Athens. Greece
		0.63	Basle. Switzerland
		0.59	Helsinki. Finland
		0.61	Prague. Czech
	Wichmann et al. [53]	0.55	Stockholm, Sweden
	Diapouli et al. [43]	0.71	Athens, Greece
	Calculated according to literature data	0.69	China
		0.32	China

**Table 3:** Parameters used to evaluate the effects on human health of indoor exposure to particulates of outdoor origin.

		PM_10_ infiltration factor	PM_2.5_ infiltration factor	T_out_ (h)
United States	Mean	0.55	0.58	1.8
	max	0.60	0.82	2.7
	min	0.51	0.45	0.9
Europe	Mean	0.36	0.63	1.8
	max	0.56	0.71	2.7
	min	0.17	0.55	0.9
China	Mean	0.36	0.51	1.6
	max	0.45	0.69	2.4
	min	0.29	0.32	0.8
Global	Mean	0.38	0.59	1.7
	max	0.60	0.82	2.7
	min	0.17	0.32	0.8

### Duration of exposure

The time spent by U.S. individuals in outdoor and indoor environments was determined from the National Human Activity Pattern Survey (NHAPS). NHAPS is a nationally representative survey (n = 9,386) conducted between September 1992 and October 1994 [24]. Based on self-reported time-activity budgets, people in the U.S. spend an average of 1.8 h outdoors per day [24]. Due to the lack of data on daily activity patterns for Europe, we extrapolated the results from the NHAPS to European populations.

Zhou and Zhao [44] reviewed time-activity patterns for both urban and rural Chinese residents and fitted log-normal distributions. The fitted distributions were then repeatedly sampled to generate a set of mean standard deviation (SD) pairs that could match the mean SD pattern of all individual studies in a mean SD plot. According to this analysis, Chinese adults spend an average of 1.6 (0.6, SD) hours outside per day.

No reports on global time-activity patterns were found in the literature, so we used 1.7 h, which is the mean of the values reported for the U.S. and China.

Sensitivity analysis was conducted to study the effect of time-activity pattern on mortality, which used a ±50% range of the mean time spent outside (see Table 3).

## Results and Discussion

**Fig 1:**
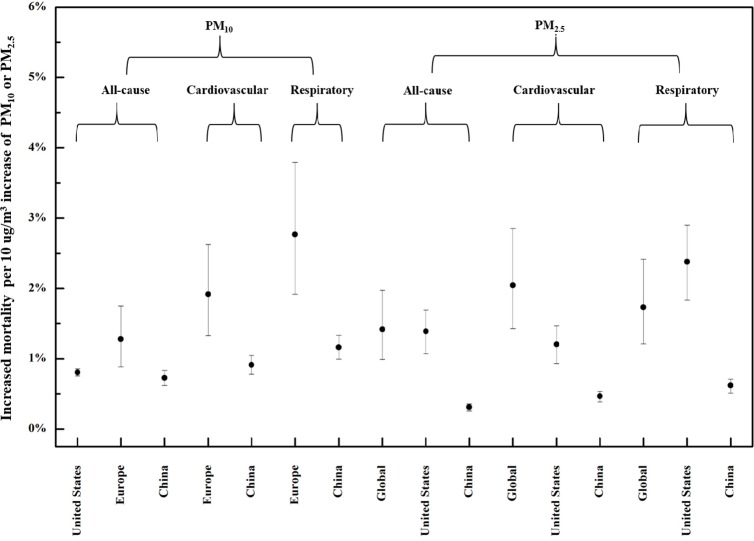
Mortality attributable to indoor exposure to particulates of outdoor origin.

Fig 1 shows the increased mortality attributable to each 10 μg/m^3^ increase in indoor exposure to PM of outdoor origin. Each 10 μg/m^3^ increase in outdoor PM_10_ is predicted to result in 0.81%, 1.20%, and 0.73% increase in all-cause mortality in the U.S., Europe, and China, respectively. The mean increases in cardiovascular mortality are 1.8% and 0.91% for Europe and China respectively, whereas those for respiratory mortality are 2.60% and 1.16%. For each 10 μg/m^3^ increase in outdoor PM_2.5_, all-cause mortality is predicted to increase by 1.29%, 1.41%, and 0.32% in the globally, U.S. and China, compared with 1.86%, 1.22%, and 0.48%, respectively, for cardiovascular mortality, and 1.57%, 2.41% and 0.65% for respiratory mortality. Overall, the predicted increases in all-cause, cardiovascular, and respiratory mortality in China are considerably lower than in other regions.

For PM_10_, the predicted increases in all-cause, cardiovascular, and respiratory mortality were all higher in Europe than in the U.S. Although the infiltration factor for PM_10_ in European countries was lower than in the U.S. and China in this study, the total increase in mortality is actually predicted to be higher in Europe, which dominates the influence on total increased mortality.

**Fig 2:**
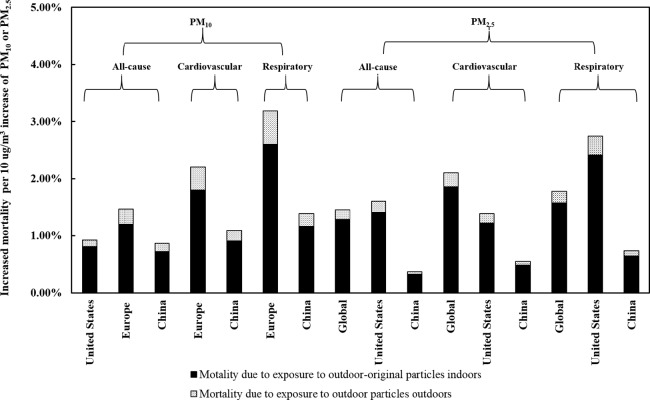
Comparison of mortality due to direct exposure to outdoor particles versus indoor exposure to particulates of outdoor origin.

Fig 2 shows increased mortality attributed to direct exposure to outdoor particles for every 10 μg/m^3^ increase in PM pollution, compared with that predicted for indoor exposure to outdoor-originated PM. Increased mortality resulting from indoor exposure accounts for 81–89% of the total increase in mortality.

The results of this study suggest that it is important to account for indoor exposure to outdoor-originated particles in the relationship between air pollution and health. PM infiltration and time-activity patterns are both important factors in human exposure, so a sensitivity analysis was conducted to determine their effects on mortality.

### Influence of infiltration

Since particle infiltration differs even between studies conducted in the same region, we conducted an extensive review of the literature, from which infiltration values are shown in Table 2. We selected the maximum and minimum infiltration values for use in the sensitivity analysis, shown in Fig 1.

For each 10 μg/m^3^ increase in outdoor PM_10_ a range of indoor infiltration values is calculated; based on these data, there is no remarkable increase in all-cause mortality associated indoor exposure in the U.S. and China (increase of 0.10–0.21%); however, all-cause mortality in Europe showed a much larger increase of approximately 0.87%. Similarly to the findings for all-cause mortality, considerable increases were observed in mean cardiovascular and respiratory mortality for Europe but not for China. For each 10 μg/m^3^ increase in outdoor PM_2.5,_ all-cause, cardiovascular, and respiratory mortalities due to indoor exposure all showed little increase in mean mortality in China; in the U.S, all-cause and cardiovascular mortalities due to indoor exposure in mean mortality increased 0.62% and 0.54%, respectively, the exception was respiratory mortality in the U.S., which showed increases of 1.07% in respiratory mortality. The each 10 μg/m^3^ increase in outdoor PM_2.5,_ all-cause, cardiovascular, and respiratory mortalities due to indoor exposure increase 0.98%, 1.42% and 1.20% respectively for the global region, which is large compare with U.S. and China. There are two explanations for this finding. One is that the mortality values of all three forms of mortality are very small, and are therefore not sensitive to the infiltration value; another is that the infiltration we reviewed in the literature is relatively comprehensive, whereas the infiltration values in the different studies differ slightly.

### Influence of exposure time

**Fig 3:**
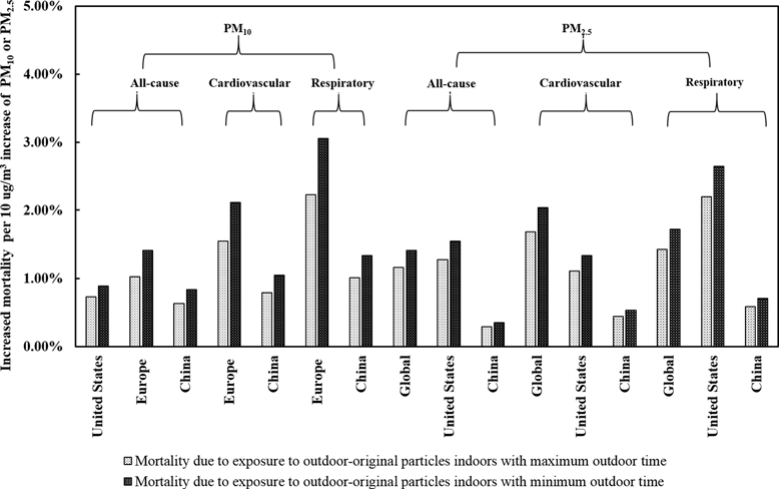
Comparison of mortality due to indoor exposure to particles of outdoor origin, according to maximum/minimum duration of outdoor exposure.

Since personal activity patterns can be highly variable, the exposure duration in indoor and outdoor environments may show frequent fluctuation. We therefore varied the duration of outdoor activity within a range of 50% when conducting the sensitivity analysis. The results are shown in Fig 3.

For each 10 μg/m^3^ increase in outdoor PM_10,_ changes in the duration of outdoor exposure are associated with remarkable changes in all-cause mortality in the U.S., Europe, and China, respectively, which derive from a reciprocal shift in the duration of indoor exposure. The mean increases in all-cause mortality associated with indoor exposure range from 0.73% to 0.89%, 1.03% to 1.41%, and 0.63% to 0.84% for the United States, Europe, and China, respectively. The mean increases in cardiovascular mortality range from 1.54% to 2.11%, and from 0.79% to 1.05% for Europe and China respectively. The mean increases in respiratory mortality range from 2.23% to 3.05%, and from 1.01% to 1.34% for Europe and China respectively.

For each 10 μg/m^3^ increase in outdoor PM_2.5_, the change in duration of outdoor exposure also shows a remarkable increase in all-cause mortality and respiratory and cardiovascular mortality associated with indoor exposure. Mean all-cause, cardiovascular and respiratory mortality due to indoor exposure to outdoor-originated PM_2.5_ show approximately increases of 0.24%, 0.35% and 0.30% globally and 0.26%, 0.22% and 0.44% for the United States when outdoor activity time decreased by two-thirds. However, when outdoor activity time decreases by two-thirds, all-cause, cardiovascular and respiratory mortality due to indoor exposure to outdoor-originated PM_2.5_ in China increase no more than 0.12% for each 10 μg/m^3^ increase in outdoor PM_2.5_.

These results indicate that outdoor-derived PM has considerable effects on human health arising from exposure within the indoor environment. Enhancing the capacity of buildings to exclude outdoor particles, and the installation of air purifiers in indoor environments are both important measures for protection of public health.

### Limitations

This study has several limitations. Since most studies on the effects of outdoor particles on human health were conducted in the U.S., Europe, and China, the data used in our models may not be representative of all regions of the world, especially Africa, South America, and Oceania, where almost no data are available the published references.

This study was also limited by a lack of infiltration factor data for much of Asia (except China), Africa, South America, and Oceania in our study (work sites and bars/restaurants). However, since detailed analysis of measured data shows that the infiltration factor is not strongly region-dependent [21–23,37–38,41–42,45–46,48–49,52,54–57], the assumed infiltration factors used in this study are not likely to deviate significantly from the actual measurements. Rare infiltration factors were studied at the same time for most of the epidemiologic studies, so further study need to be conducted to obtain the detailed and systematic infiltration factors. This is also what we want to do in the future work. For accurately estimate as possible, we conducted the literature review to determine the PM_10_ and PM_2.5_ infiltration factors for European, North American, Chinese, and global residences, try to apply local infiltration factors we could obtain for the corresponding regions (see Table 2). We also calculated mean infiltration factors of PM_10_ and PM_2.5_ in each region, and the maximum and minimum values for each region were further used in sensitivity analysis to study the effect of infiltration factor on mortality and particle infiltration. We think such treatment of infiltration factors represents the state of the art approach for such study.

Parameters such as particle size, the crack geometry of building envelopes, differences in indoor/outdoor air pressure, and the efficiency of mechanical filtration can affect particle infiltration; however, such factors are highly complex and require further study. After analyzing these factors, we were able to account for the relationship between mortality and indoor exposure to PM of outdoor origin.

Finally, although Daniels et al. [29] concluded that log-linear models are appropriate for assessing the effect of PM pollution on daily mortality, and many studies have employed such models to analyze epidemiological data [6–7, 29–31], the exact nature of this relationship has not been conclusively established. The assumption of linearity is a potential limitation of the model applied in this study, and warrants further research.

### Perspectives

The limited scientific knowledge on the health effects of exposure to airborne particles in the indoor environment represents a major barrier to establishing limit values or guidelines that protect public health [58]. One explanation for the lack of studies on the health effects of indoor exposure to outdoor-originated PM is the expense, in addition to the logistical and technological constraints inherent in measuring direct personal PM exposure. This study introduces an innovative method for quantifying the relationship between outdoor PM and indoor PM originating from outdoors, based on a known set of physical principles. For example, based on existing knowledge of aerosol physics, we know that this relationship is affected by the particle penetration factor through building envelopes, in conjunction with the rates of indoor particle deposition and air exchange. Reducing the need for conducting personal PM exposure measurements may facilitate further studies on this topic so that guidelines on indoor exposure to ambient PM can be established.

This method for estimating mortality derived from indoor exposure to particles of outdoor origin has some disadvantages (e.g., the composition issue). Wilson et al. [28] suggested a reasonable, epidemiological approach to estimate such mortality. For the purpose of controlling aerosol particle pollution, we are eager to learn from the epidemiological data, e.g. mortality derived from indoor exposure to particles of outdoor origin, from the method suggested by Wilson et al. [28] in order to support strategies for managing indoor air quality. However, to date, no such studies have been presented. Therefore, through this paper, we also wish to encourage epidemiologists to further consider this important issue.
